# Motivation and Intention Toward Physical Activity During the COVID-19 Pandemic: Perspectives From Integrated Model of Self-Determination and Planned Behavior Theories

**DOI:** 10.3389/fpsyg.2021.714865

**Published:** 2021-07-29

**Authors:** Dojin Jang, Inwoo Kim, Sungho Kwon

**Affiliations:** ^1^Department of Physical Education, College of Education, Seoul National University, Seoul, South Korea; ^2^Department of Sports Culture, College of the Arts, Dongguk University, Seoul, South Korea

**Keywords:** COVID-19, physical activity, basic psychological needs, self-determined motivations, social cognitive beliefs

## Abstract

**Background:** In attempts to hinder the spread of the Coronavirus disease 2019 (COVID-19), many countries have continued distancing, isolation, and quarantine measures, which has led to limited opportunity of physical activity. This study provides empirical support for a motivational process behind physical activity during the COVID-19 pandemic by testing the influence of psychosocial variables derived from the integrated model of self-determination theory (SDT) and the theory of planned behavior (TPB).

**Methods:** A cross-sectional survey was conducted among Korean adults (*N* = 248). Participants completed the measures of SDT and TPB constructs modified to reflect their participation in physical activity during the COVID-19 pandemic. A sample size of 243 participants was employed, and the integrated model was tested using serial multiple mediation analysis to check the hypothesized relationships.

**Results:** Findings indicated that in the COVID-19 context, the satisfaction of basic psychological needs positively predicts the level of self-determined motivations for physical activity, which is partially related to the level of social cognitive beliefs and intentions. The findings also demonstrated that attitude toward physical activity during COVID-19 was a major variable explaining the serial multiple relationships between the SDT and TPB constructs. The potential influence of demographics (gender, age, marital status, and past physical activity) was controlled as a covariate, and no significant effects were identified.

**Conclusion:** The current study identified the psychosocial mechanisms of intention of South Koreans' physical activity during the COVID-19 pandemic, which could be used as an empirical basis for the development of interventions to maintain or strengthen physical activity in unprecedented situations.

## Introduction

The coronavirus disease 2019 (COVID-19) pandemic has introduced many unexpected changes in our lives and poses a direct threat to life (Arora and Grey, 2020; Van Bavel et al., 2020; Venkatesh, 2020). As measures to control the spread of the virus, national shut down and lock down policies such as border closure, movement restrictions, travel ban, and home quarantine are becoming the “new normal” in our society (Bates et al., 2020). While such responses to COVID-19 are evaluated to be effective as non-pharmaceutical methods to prevent or mitigate the spread of the basic reproduction number (R_0_) of the virus (Min et al., 2020), they raise concerns about the limited opportunity of physical activity necessary to maintain personal health and prevent disease (Matias et al., 2020). In fact, the physical activity patterns of people have changed significantly (Tison et al., 2020), and nearly 4 billion people around the world reportedly experience social isolation (Sandford, 2020). This could last 1–2 years worldwide until the end of the pandemic (López and Rodó, 2020; Matias et al., 2020).

The Global Recommendation on Physical Activity for Health by the World Health Organization (2019) recommends that adults aged 18–64 or older, engage in at least 150 min of moderate physical activities a week or at least 75 min of high-intensity physical activities a week, for health benefits. However, one-third of the adult population does not appear to meet these recommended guidelines. The same is true in South Korea (Ministry of Culture, Sports, and Tourism of The Republic of Korea, 2019), and restrictions introduced due to COVID-19 (distancing/isolation/quarantine) could worsen this situation (Kim et al., 2020). According to a survey by a South Korean government agency, 67.8% of citizens agreed to the preventive measures for COVID-19. The ratio of objections to multi-use facilities and events was ~50%, raising concerns about the reduction in physical activity in general (Lee and You, 2020).

Although the level differs according to demographic variables, the spread of COVID-19 and subsequent distancing/isolation/quarantine measures have influenced the actual decrease in the level of physical activity of people (Lesser and Nienhuis, 2020; Moore et al., 2020; Romero-Blanco et al., 2020). A reduction in physical activity due to inevitable restrictions can increase vulnerability to the COVID-19 infection itself and can lead to personal health risks, such as cardiovascular disease, and social problems, such as increased medical expenses (Carter et al., 2020; Narici et al., 2020; Woods et al., 2020). In addition, restricted outdoor activities and suppressed social interactions could affect mental health concerns, such as stress, depression, and anxiety (Brooks et al., 2020; Maugeri et al., 2020; Meyer et al., 2020). Consequently, maintained participation in physical activity amid the physical/social constraints due to COVID-19 must be considered for the physical and mental health of the public and individuals (Chtourou et al., 2020; Sallis et al., 2020).

Despite these adverse conditions, there have been continuous social and individual efforts to promote and participate in physical activity. Governments, sports, or exercise-related organizations present guidelines for improving the physical activity of people during the pandemic (see Dwyer et al., 2020). Home-training (Kaushal et al., 2020), social media (Hayes, 2020), and virtual reality (Gao et al., 2020) are attracting attention as a new normal for sports participation and physical activity. Even in the midst of anxiety, there are people who continue to engage in sports and exercise as usual, or even strengthen their routine (Brand et al., 2020; Rhodes et al., 2020; Cheval et al., 2021; Mutz and Gerke, 2021). Several studies have shown that participation in such physical activity is an effective response to the regulation of the immune system against infectious diseases (Pedersen and Saltin, 2015). Importantly, what psychological characteristics would those who remain physically active despite the limited COVID-19 conditions, exhibit? This study aims to shed light on this question using a theoretical model of exercise psychology.

**Figure 1:**
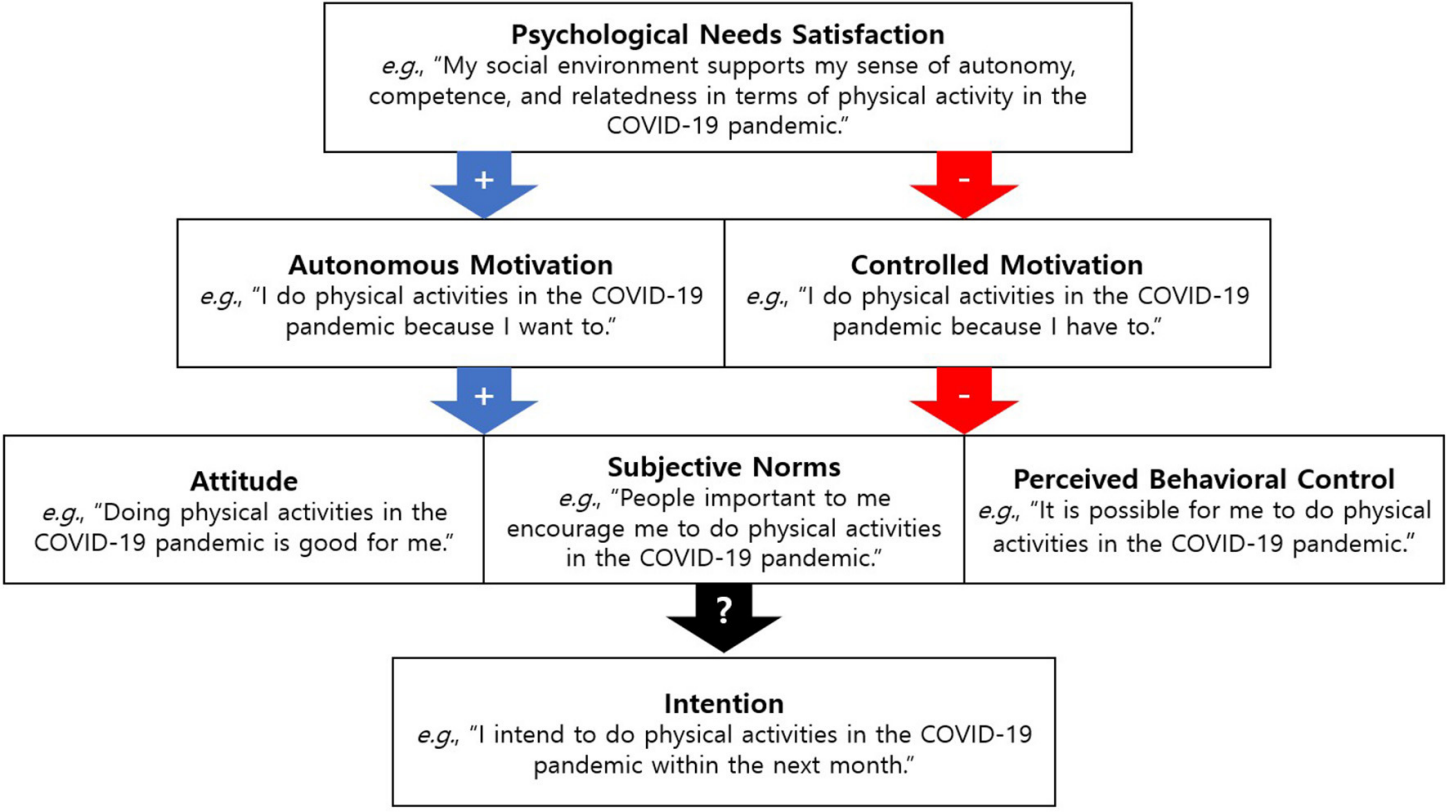
The integrated model of Self-Determination Theory and Theory of Planned Behavior.

Basic research is required to comprehensively draw socioecological and psychological factors affecting the behavior of individuals at a time of unexpected large-scale behavioral change (Venkatesh, 2020). The integrated model of self-determination theory (SDT) and theory of planned behavior (TPB), is a behavioral change model that uses two major theoretical concepts of psychology (Hagger et al., 2002a,b; Hagger and Chatzisarantis, 2009, 2016; Figure 1). This integrated model explains various health behaviors, such as drinking and smoking, sexual behaviors, as well as physical activity, and has also been successfully applied in various cultures (Hagger and Chatzisarantis, 2009, 2016). According to this integrated model, the types of support for psychological needs and the corresponding motivations explain social and cognitive beliefs, intentions, and behaviors. In other words, when social and environmental factors support psychological needs (autonomy/competence/relatedness), autonomous motivation increases, which further increases the likelihood of continuing the target behavior (physical activity and exercise) with more favorable beliefs (attitude/subjective norm/perceived behavioral control) and intentions. Conversely, when these needs are strictly controlled, the intention and duration of such behaviors can diminish.

Chan et al. (2021) proposed this model as a theoretical framework for predicting preventive behaviors of COVID-19, but it can also be utilized to explain physical activity emphasized during the COVID-19 pandemic. Empirical evidence suggests that this model significantly predicts physical activity (Brooks et al., 2017; Hamilton et al., 2018). Consequently, this study uses the integrated model of SDT and TPB as a theoretical framework to identify psychosocial factors associated with physical activity under the policy restrictions due to the pandemic. The results could provide an empirical basis for the development of interventions to maintain or strengthen physical activity in unprecedented situations.

### Present Research

This study aims to explain the motivational mechanism of physical activity during the COVID-19 pandemic based on the integrated model of SDT and TPB. Predicting the intention of physical activity during the COVID-19 situation can begin with the satisfaction of basic psychological needs in that context. This relationship can be mediated by the level of self-determined motivation and the corresponding social and cognitive beliefs. The integrated SDT and TPB beliefs may be related to the theoretical background; the mutual, structural relationship will enhance physical activity predictions during the COVID-19 pandemic. Based on the theoretical background of the integrated model, the research hypotheses are as follows.

*H1: The satisfaction of basic psychological needs during COVID-19 can predict the level of self-determined motivation for physical activity*.

*H2: The level of self-determined motivation during COVID-19 can predict social and cognitive beliefs and the intention to engage in physical activity*.

*H3: The relationship between satisfaction of basic psychological needs and intention for physical activity during COVID-19 can be mediated by self-determined motivation and social and cognitive beliefs*.

## Methods

### Study Area

A cross-sectional web-based survey was conducted to examine the proposed hypotheses. This survey was conducted in South Korea between September and November 2020. At the time of data collection, the COVID-19 pandemic and consequential lockdown was in its enhanced phase in Korea, with ~5,000 confirmed cases per month (Kim and Kim, 2020). The online survey was the most feasible way to access the target population considering the social distance protocols implemented during the COVID-19 pandemic.

The target population was adults aged 19 years and older (Korea's civil law) residing in South Korea for more than 6 months since March 12, 2020, when the WHO declared the COVID-19 pandemic. To reflect a representation of South Korea while considering regional differences, Korean adults who have lived in the Seoul metropolitan area (including the Seoul, Gyeonggi-do, and Incheon areas, in which 50.0% of the Korean population resides as of 2020) were selected. More than half of confirmed cases of COVID-19 occurred in this area.

### Procedures and Participants

All procedures for the current study were approved by the Institutional Review Board of Seoul National University (No. 2010/003-009), Seoul, South Korea. Using the non-probabilistic snowball sampling method, the link to the online survey was disseminated via social media (e.g., Facebook, Instagram, and Kakao Talk), and shared with the personal contacts of the research team members. The survey invitation containing general information about the survey, such as its aim, consent statement, and participant incentive (KRW 5000 [is about US $4]) was provided on the first page of the online survey. Respondents provided consent by clicking “Agree to participate in this survey” on the same page, before moving on to answer the survey questions. The survey took ~25 min to complete. Only anonymized data were stored and analyzed.

Using G-power 3.1.9.7, α = 0.05, effect size (*f*^2^) = 0.15 (medium), and number of predictors = 5 were applied. Consequently, a sample size of 138 was required in the analysis of the current study (linear multiple regression). However, the current study included a greater number of subjects to cope with the possibility of non-response errors (Howell, 2004). To minimize the impact of errors due to this type of data, we adopted a cleaning process that consisted of the following steps: removal of ineligible cases and multiple submissions from the same respondent, and identification and handling of meaningless data. The latter was represented by invalid responses to the questionnaire due to the lack of internal consistency of responses. Overall, 248 Korean adults had access to the self-administrated survey, and 243 valid respondents were included in the following procedure. The demographics of the participants are presented in Table 1.

**Table 1:** Demographics of the participants (*n* = 243).

**Demographics**	**Frequency**	***%***
**Gender**
Male	169	69.5
Female	74	30.5
**Age**
19 ~ 29 yrs.	143	58.8
30 ~ 39 yrs.	87	35.8
40 ~ 60 yrs.	13	5.4
**Marital status**
Single	106	43.6
With family/partner	137	56.4
Exercise volume^*^
Pre COVID-19 pandemic		
0 ~ 60 min.	68	28.0
61 ~ 149 min.	53	21.8
150 min. ~	122	50.2
Post COVID-19 pandemic
0 ~ 60 min.	130	53.5
61 ~ 149 min.	42	17.3
150 min. ~	71	29.2

* Exercise volume was calculated by sum (time * frequency) of moderate to vigorous physical activity per week.

### Measurements

Using the Google online survey platform, a new questionnaire was developed, after validation by two experts (authors KIM and KWON), who were working as physical educationists as well as sports and exercise psychologists with more than 10 years of experience. The online self-reporting questionnaire consisted of four parts: demographics, basic psychological needs, self-determined motivation, and TPB constructs. Demographic variables included gender, age, marital status, and the volume of exercise before and after the COVID-19 pandemic, measured using the recall method. These personal characteristics are considered to be related to the SDT and TPB mechanisms in the context of physical activity (Hagger and Chatzisarantis, 2009, 2016). In addition, the items of psychological characteristics were based on commonly used, previously validated scaled measures adopted from previous research (in the following sections). The measurements for these items were adjusted to a 6-point Likert scale to prevent median bias (Abdul, 2010).

#### Basic Psychological Needs Satisfaction (BPNS)

The Psychological Need Satisfaction in Exercise Scale (PNSE; Wilson et al., 2006) was used to measure the basic psychological needs for physical activity participation and adherence. The PNSE has a total of 18 questions, including six items each for autonomy, competence, and relatedness. The psychometrics of the Korean version of the scale were validated (Jeong and Kim, 2015). In the current study, questions was modified to reflect the psychological needs related to participating in physical activities during the COVID-19 pandemic (e.g., Autonomy: I feel like I am capable of doing even the most challenging exercises during the COVID-19 pandemic; Competence: I feel like I am in charge of my own exercise program decisions during the COVID-19 pandemic; and Relatedness: I feel a sense of camaraderie with my exercise companions because we exercise for the same reasons during the COVID-19 pandemic). A higher total response score indicated a greater level of BPNS for physical activity in COVID-19 situations (0 = “not at all” and 5 = “very true”).

#### Self-Determined Motivation

The Behavioral Regulation in Exercise Questionnaire-2 (BREQ-2; Markland and Tobin, 2004) was applied to measure autonomous motivation (AM)/controlled motivation (CM) for participation in physical activity. BREQ-2 reflects the five behavioral regulation styles presented in the SDT (Ryan et al., 2009), consisting of a total of 5 factors (Amotivation, External regulation, Introjected regulation, Identified regulation, and Intrinsic motivation) and 19 items (0 = “not at all” and 5 = “very true”). The construct validity of the Korean version of the scale was supported by Hwang and Kim (2013). Each of the four items of External Regulation and Intrinsic motivation were modified to represent two types of motivation (AM and CM) for participation in physical activity during the COVID-19 pandemic (e.g., during the COVID-19 pandemic, I (will) participate in physical activity, because. AM: I find exercise a pleasurable activity, and CM: I feel under pressure from my friends/family to exercise). To ensure the simplicity of the model in the current study, the relative autonomy index (RAI; Grolnick and Ryan, 1987) was calculated (AM^*^ [+2]; CM^*^ [−2]). Higher RAI scores indicated greater levels of self-determination with respect to physical activity during COVID-19 situations (minimum: −10 to maximum: 10).

#### TPB Constructs

Items related to TPB and exercise participation used in the study by Kim and Cheon (2018), were used to measure social cognitive beliefs and the intention to participate in physical activity. The items were developed based on TPB (Ajzen, 2002). These concepts have been evaluated as significant for predicting health-related behaviors (Hagger et al., 2016). In the current study, each of four items: Attitude (ATT), Subjective Norms (SN), Perceived Behavioral Control (PBC), and Intention (INT), were modified to measure the cognitive belief level and intention to participate in physical activity during the COVID-19 pandemic (e.g., ATT: Exercising during the COVID-19 pandemic is valuable and meaningful to me; SN: most others support me to exercise during the COVID-19 pandemic; PBC: I feel in complete control over whether I will exercise during the COVID-19 pandemic; INT: I will participate in various physical activities within the next month). The item scores of each construct are aggregated into a single score, which is operationally defined as the level of each belief and intention (0 = “not at all” and 5 = “very true”).

### Data Analysis

Using IBM SPSS (Statistics 25.0 and Amos 23.0, IBM Corp., Armonk, NY, USA) and PROCESS 3.5 for SPSS (Hayes, 2018), statistical analyses were performed to validate the collected data and research models following these procedures: First, descriptive statistics (mean, standard deviation, skewness, and kurtosis) were calculated to verify the normality of the collected data. Second, to verify the validity of each measurement tool (questionnaire), a confirmatory factor analysis (CFA) with maximum likelihood (ML) estimation and an internal consistency analysis (Cronbach's α) were conducted, which supported a set of items parceled for each questionnaire. Third, a CFA with ML estimation was conducted to verify the construct validity of the entire measurement model, along with a check for the goodness-of-fit index (χ^2^, comparative fit index [CFI], Tucker–Lewis index [TLI], root mean square error of approximation [RMSEA]; Hu and Bentler, 1999), construct reliability (*CR*), and average variance extracted (*AVE*), for each factor (Hair et al., 2010). Fourth, serial multiple mediation analysis (Model 81 as described in PROCSESS; Hayes, 2018) with bootstrapping (*n* = 5,000) was performed to test the research model, controlling for the potential influence of demographics (gender, age, marital status, and past physical activity) as covariates. The statistical significance for all analyses was set at α = 0.05.

## Results

### Measurement Tools CFA

The univariate normality of an item was assumed if its skewness and kurtosis values ranged between −1.96 and +1.96 (Kim, 2013). The preliminary analyses indicated that all the dimensions of the items achieved univariate normality; skewness values ranged from −1.199 to 1.157, and kurtosis values ranged from −1.332 to 0.975.

The results of CFA and Cronbach's α for each measurement tool (questionnaire) are as follows:

The second-order CFA for PNSE showed satisfactory fit for the data, except for nine items (Autonomy: Items 4, 5, 6; Competence: Items 1, 4, 6; and Relatedness: Items 1, 4, 6) with a factor loading lower than 0.70 (χ^2^ = 56.194, *df* = 24, *p* < 0.001, TLI = 0.977, CFI = 0.985, RMSEA = 0.074). The Cronbach's α for PNSE was shown to be the overall BPNS (9 items) = 0.935 with the sub-factors of autonomy (three items) = 0.896, competence (three items) = 0.939, and relatedness (three items) = 0.935. Since the higher-order factor structure was supported, the total BPNS score was utilized in subsequent analyses (Sánchez-Oliva et al., 2017).

The first-order CFA for BREQ-2 showed an acceptable fit for the data, except for two items (AM: Item 1; CM: Item 3) with a factor loading lower than 0.70 (χ^2^ = 25.290, *df* = 8, *p* = 0.001, TLI = 0.963, CFI = 0.980, RMSEA = 0.095). Cronbach's α for BREQ-2 was shown to be the sub-factor of AM (3 items) = 0.928 and CM (3 items) = 0.825. The average score for both factors was aggregated into the RAI and utilized in subsequent analyses.

The CFA for the TPB constructs (Social Cognitive Beliefs and Intention) showed an acceptable fit for the data except for one item (INT: Item 4) with a factor loading lower than 0.70 (χ^2^ = 255.682, *df* = 84, *p* < 0.001, TLI = 0.926, CFI = 0.941, RMSEA = 0.092). Cronbach's α for the TPB model was shown to be the sub-factor of ATT (4 items) = 0.919, SN (4 items) = 0.882, PBC (4 items) = 0.879, and INT (3 items) = 0.896. Based on the above results, the item parceling averaging the score for each sub-factor was applied to the subsequent analysis (Little et al., 2013).

### Measurement Model CFA

The CFA for the entire measurement model, including seven latent variables and 30 observational variables, showed satisfactory goodness-of-fit (χ^2^ = 788.211, *df* = 381, *p* < 0.001, TLI = 0.927, CFI = 0.936, RMSEA = 0.066). All factor loadings (0.728~0.951) for the items in the measurement model were significant (*p* < 0.001), which means that the latent variable included in the model explains the data well (see Appendix).

Table 2 shows descriptive statistics (*M, SD*), bivariate correlations, and a convergent/discriminant validity index (*CR, AVE*, and *MSV*) of latent variables. The correlation between variables was generally significant except for some values with CM. BPNS and PBC showed the highest correlation, and PBC had the highest correlation with the dependent variable INT, followed by ATT, BPNS, SN, and AM. In addition, *CR* and *AVE* values supported the convergent and discriminant validity of the measurement model: *CR* = 0.748–0.940 (≥ 0.70), *AVE* = 0.617–0.813 (≥ 0.50), and each *AVE* > each *MSV*.

**Table 2:** Means, standard deviations, bivariate correlations, and model validity measures.

***Variables***	**BPNS**	**AM**	**CM**	**ATT**	**SN**	**PBC**	**INT**	*M*	*SD*
**BPNS**	**0.898**							2.83	1.28
**AM**	0.461^***^	**0.902**						3.25	1.19
**CM**	0.126	0.133	**0.785**					1.26	1.56
**ATT**	0.618^***^	0.500^***^	0.097	**0.861**				3.53	1.30
**SN**	0.514^***^	0.332^***^	0.374^***^	0.515^***^	**0.814**			2.61	1.39
**PBC**	0.733^***^	0.474^***^	0.219^**^	0.687^***^	0.641^***^	**0.805**		2.86	1.36
**INT**	0.674^***^	0.425^***^	0.145	0.676^***^	0.528^***^	0.698^***^	**0.879**	3.41	1.33
*CR*	0.940	0.914	0.748	0.896	0.838	0.827	0.887	–	–
*AVE*	0.806	0.813	0.617	0.743	0.662	0.648	0.772	–	–
*MSV*	0.537	0.224	0.140	0.472	0.411	0.537	0.454	–	–

*Note 1. The construct labels are as follows: BPNS, Basic Psychological Needs Satisfaction; AM, Autonomous Motivation; CM, Controlled Motivation; ATT, Attitudes; SN, Subjective Norms; PBC, Perceived Behavioral Control; INT, Intention; M, Mean; SD, Standardized Deviation; CR, Construct Reliability; AVE, Average Variance Extracted; MSV, Maximum Shared Variance*.

*Note 2. Bold values in the diagonal represent square root of AVE*.

** *p < 0.01*,

*** *p < 0.001*.

### Serial Multiple Mediation Analyses for SDT and TPB

**Figure 2:**
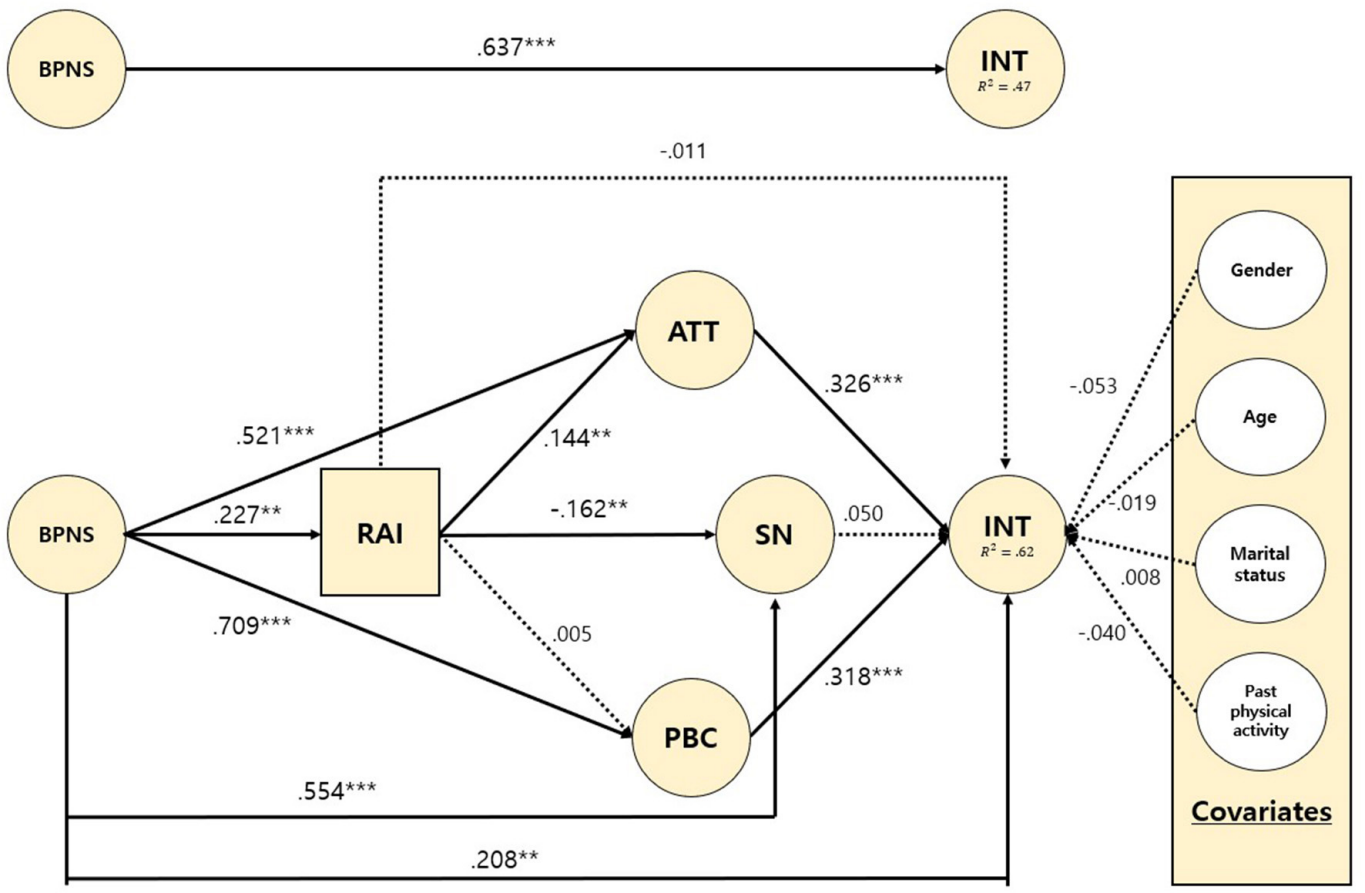
Direct and mediation model with path coefficients. Note 1. The construct labels are as follows: BPNS, basic psychological needs satisfaction; RAI, relative autonomy index; ATT, attitudes; SN, subjective norms; PBC, perceived behavioral control; INT, intention. Note 2. The covariate codes are as follows: Gender = male (1), female (2); Age (yrs) = continuous (19~60); Marital status = Single (1), With family/partner (2); Past physical activity (min.) = continuous (0~1260). Note 3. All statistics represent direct relationship between variables. **p < 0.01, ***p < 0.001.

Table 3, Figure 2 show the serial multiple mediating effects of RAI and social cognitive beliefs (ATT/SN/PBC) in the relationship between BPNS and INT (gender, age, marital status, and past physical activity were controlled as covariates).

**Table 3:** Path coefficients of direct effects for mediation model.

***Path***	**B**	**β**	***SE***	***t***	***Model-fit***
BPNS → **RAI**	0.548	0.227	0.164	3.331^**^	*R* = 0.091
					*F*_(5, 237)_ = 4.739^***^
BPNS → **ATT**	0.530	0.521	0.055	9.665^***^	*R* = 0.458
RAI → **ATT**	0.061	0.144	0.021	2.868^**^	*F*_(6, 236)_ = 33.209^***^
BPNS → **SN**	0.603	0.554	0.067	9.035^^***^^	*R* = 0.297
RAI → **SN**	−0.073	−0.162	0.026	−2.837^**^	*F*_(6, 236)_ = 16.619^***^
BPNS → **PBC**	0.755	0.709	0.053	14.337^^***^^	*R* = 0.544
RAI → **PBC**	0.002	0.005	0.020	0.100	*F*_(6, 236)_ = 46.908^***^
BPNS → **INT**	0.216	0.208	0.067	3.198^**^	*R* = 0.618
RAI → **INT**	−0.005	−0.011	0.019	−0.246	*F*_(9, 233)_ = 41.918^***^
ATT → **INT**	0.332	0.326	0.060	5.505^^***^^	
SN → **INT**	0.048	0.050	0.054	0.877	
PBC → **INT**	0.310	0.318	0.066	4.662^^***^^	

*Note 1. The construct labels are as follows: BPNS, Basic Psychological Needs Satisfaction; RAI, Relative Autonomy Index; ATT, Attitudes; SN, Subjective Norms; PBC, Perceived Behavioral Control; INT, Intention*.

*Note 2. The covariate codes are as follows: Gender = male (1), female (2); Age (yrs) = continuous (19~60); Marital status = Single (1), With family/partner (2); Past physical activity (min.) = continuous (0~1260)*.

*Note 3. All statistics represent direct relationship between variables*.

** *p < 0.01*,

*** *p < 0.001*.

#### Testing the Direct Effect

Table 3 indicates the direct path from the predictors. First, BPNS had a significantly positive effect on RAI, ATT, SN, PBC, and INT. Second, RAI had a significantly positive effect on ATT, but a negative effect on SN, and no significant effect on PBC and INT. Third, for the dependent variable (INT), ATT, and PBC had a significantly positive effect, but SN had no significant effect. This partially supports *H1* and *H2*.

In addition, as seen in Figure 2, the direct effect of the independent variable (BPNS) on the dependent variable (INT) decreased when mediators (RAI and ATT/SN/PBC) were included in the model (β = 0.637, *p* < 0.001 → β = 0.208, *p* < 0.01). In the full model, the amount of explanation for the INT variance from the predictor variables increased from 47% (*R*^2^ = 0.471) to 62% (*R*^2^ = 0.618). These results indicate that RAI and ATT/SN/PBC partially mediate the relationship between BPNS and INT under the basic assumptions of Baron and Kenny (1986). Moreover, this result could be interpreted under the condition in which gender (β = −0.053, *p* = 0.243), age (β = −0.019, *p* = 0.672), marital status (β = 0.008, *p* = 0.862), and past physical activity (β = −0.040, *p* = 0.355) were controlled. *H3* is partially supported.

#### Testing the Indirect Effect

Table 4 shows the (total/specific) indirect effects of RAI and social cognitive beliefs (ATT/SN/PBC) by applying bootstrapping (*n* = 5,000) with 95% confidence intervals. First, the total indirect effect was significant (B = 0.445, 95% C.I = 0.329~0.560). Second, the simple mediation path of BPNS → ATT → INT (B = 0.176, 95% CI = 0.087–0.274) and BPNS → PBC → INT (B = 0.234, 95% CI = 0.117–0.347) was significant. Third, the serial mediation path of BPNS → RAI → ATT → INT (B = 0.011, 95% CI = 0.002–0.027) was significant. These results indicate that the integrated model of SDT and TPB applied in the COVID-19 context significantly predicted the intention for physical activity, especially through the path of ATT.

**Table 4:** Path coefficients of indirect effects for mediation model.

***Path***	**B**	**Boot SE**	**Bootstrapping 95%C.I**.		
			**Lower**	**Upper**	**Significance**
**BPNS** **→** **RAI** **→** **INT**	−0.003	0.011	−0.025	0.020	Not sig.
**BPNS** **→** **ATT** **→** **INT**	0.176	0.048	0.087	0.274	Sig.
**BPNS** **→** **SN** **→** **INT**	0.029	0.038	−0.043	0.107	Not sig.
**BPNS** **→** **PBC** **→** **INT**	0.234	0.058	0.117	0.347	Sig.
**BPNS** **→** **RAI** **→** **ATT** **→** **INT**	0.011	0.007	0.002	0.027	Sig.
**BPNS** ** → RAI** **→** **SN** **→** **INT**	−0.002	0.003	−0.008	0.004	Not sig.
**BPNS** **→** **RAI** **→** **PBC** **→** **INT**	0.000	0.004	−0.008	0.009	Not sig.
*Total*	0.445	0.058	0.329	0.560	Sig.

*Note 1. The construct labels are as follows: BPNS, Basic Psychological Needs Satisfaction; RAI, Relative Autonomy Index; ATT, Attitudes; SN, Subjective Norms; PBC, Perceived Behavioral Control; INT, Intention*.

*Note 2. The covariate codes are as follows: Gender = male (1), female (2); Age (yrs) = continuous (19~60); Marital status = Single (1), With family/partner (2); Past physical activity (min.) = continuous (0~1260)*.

*Note 3. All statistics represent indirect relationship between variables*.

*Note 4. Bootstrapping n = 5,000*.

## Discussion

This study attempted to explore the social cognitive mechanism of physical activity participation in the context of COVID-19 based on the integrated model of SDT and TPB proposed by Hagger et al. (2002a,b) and Hagger and Chatzisarantis (2009, 2016). The overall study results are discussed as follows.

First, the integration of the SDT (increased level of self-determined motivation according to satisfaction of basic psychological needs) and TPB (the influence of the attitude, subjective norms, and perceived behavioral control as close variables of intention) structures was still significant in the context of COVID-19. Kaushal et al. (2020) applied this model by setting home-based physical activity as the target behavior during COVID-19 and suggested the importance of access to exercise equipment and PBC in a limited environment. Chirico et al. (2020) applied this model to the Italian population and suggested that anxiety about COVID-19 and its regional differences could be variables that regulate physical activity intention and participation. Although these studies present empirical evidence to show that physical activity during COVID-19 can be explained based on the proposed integrated model, it does not fully reflect the motivational mechanisms of SDT. In other words, the research model includes only one variable representing SDT—autonomous motivation. Ryan and Deci (2000a,b) set the social context influencing the motivation process of behavior as the premise of SDT, which indicates the satisfaction of basic psychological needs consisting of autonomy, competence, and relatedness. In the meta review by Hagger and Chatzisarantis (2009), the independent influence of needs satisfaction was emphasized as a variable preceding autonomous motivation (or self-determined motivation). This indicates that manipulation of environmental variables supporting needs satisfaction could be a direct intervention to induce changes in health behavior (Shen et al., 2007). The current study is a valuable approach that fully includes the mechanisms of SDT (needs satisfaction → self-determinant motivation) and TPB (beliefs → intention) by reflecting this basic concept.

Second, the direct pathways between the SDT and TPB constructs were discussed in comparison with earlier studies. The significantly positive effect of BPNS on RAI was consistent with the suggestion of applying SDT to physical activity (Ryan et al., 2009). The current study supported that the level of autonomy, competence, and relatedness in relation to physical activity, could predict autonomous motivation of target behavior even in the uncontrolled environment of COVID-19. This relationship was also observed among adults participating in regular physical activity during COVID-19, which indicated that a high level of BPNS and SDT predicted sports commitment (Leyton-Román et al., 2021). Next, the influence of RAI on social cognitive beliefs was positive in ATT, negative in SN, and insignificant in PBC. The positive influence on ATT was interpreted as a positive evaluation of the value of physical activity in an autonomously motivated state. The negative influence on SN was interpreted as a reduction in social pressures caused by voluntary choices (Hagger and Chatzisarantis, 2009). However, the insignificant effect of RAI on PBC appeared to reflect the limitations of physical activity in the COVID-19 situation. In addition, BPNS, ATT, and PBC were found to be variables that significantly predicted intentions. The significant positive effects of ATT and PBC were consistent with the results of the supporting TPB meta-study on health-related behaviors (McEachan et al., 2011). This indicates that positive attitudes toward physical activity and control over behavior still acted as powerful predictors of intention in the COVID-19 situation. These results could be interpreted under the condition in which gender, age, marital status, and past physical activity were controlled.

Third, gender, age, marital status, and past physical activity, which were input as covariates, did not significantly affect the hypothetical pathway of the study. However, these demographics are considered to be closely related to the SDT and TPB mechanisms in the context of physical activity (Pettee et al., 2006; Nigg et al., 2009). Empirical studies on multiple populations have also reported that changes in physical activity (frequency/intensity) during pandemics are dynamically related to demographic variables (Rhodes et al., 2020; McCarthy et al., 2021; Wilke et al., 2021). In fact, a panel study of over 45,000 UK adults showed that the overall participation in physical activity and exercise during COVID-19 tends to be low among older aged individuals, females, and those living alone (Fancourt et al., 2020). Therefore, an unexpected result in the current study may be related to insignificant gender differences in the perception of the vulnerability and severity of COVID-19 in South Korea (Lee and You, 2020). In the case of age, it may reflect the characteristics of the sample in the current study, which was biased for subjects in their 10–20 s. In particular, the level of past physical activity was reported to strongly predict future behavioral intentions and behaviors in the preceding integrated model studies (Hagger and Chatzisarantis, 2009, 2016), but it had no significant effect in the COVID-19 context. In other words, the current social cognitive (SDT and TPB) variables related to physical activity explained the intention for behavior more powerfully than the experience of physical activity before the pandemic. In fact, Maltagliati, Rebar, Fessler, Forestier, Sarrazin and Chalabaev (2021) longitudinal study revealed that physical activity during COVID-19 was strongly associated with a habit of mid-lockdown rather than before-lockdown and suggested that an autonomous motivation toward physical activity may be more important in interventions aimed at sustaining habits after a context change.

Fourth, the final research hypothesis, a serial multiple mediation model, was partially supported, and the role of ATT was particularly noted. When the influence of RAI was controlled, the relationship between BPNS and INT was mediated by ATT and PBC. When the relationship between BPNS and RAI was premised, only ATT was found to be a significant mediator. In the COVID-19 situation, BPNS for physical activity could increase INT through positive evaluation of ATT and PBC. Moreover, ATT could specify the increase in INT with an increase in the level of self-determined motivation. The ATT included in this study could be defined as an evaluation of the benefits of physical activity in the COVID-19 situation (see Appendix). Therefore, an approach that emphasizes the positive benefits of behavior would be a useful intervention in the self-determined motivation of physical activity during the COVID-19 situation. In particular, it was found that the ATT toward physical activity, which is based on health consciousness during the pandemic, could play a significant role in a healthy lifestyle, including regular physical activity (Pu et al., 2020; Sang et al., 2020). The search and identification of the practical values that constitute such an attitude remain the main tasks of a follow-up study.

### Practical Implications

Collectively, this was the first study to identify the social and cognitive mechanisms of intention to perform physical activity among South Korean adults. The findings could be used as an empirical basis for the development of interventions to maintain or strengthen physical activity during the COVID-19 situation. As revealed in this study, the attitude toward physical activity during COVID-19 was found to be a major variable explaining the serial multiple relationships between the SDT and TPB constructs. Accordingly, cultivating a positive attitude toward physical activity would be a requisite for a healthy life even during a pandemic. Personal attitudes could be built from our own personal experience or from the influence of the environment around us. Therefore, it is suggested that social and individual efforts to promote physical activity need to focus on their importance and value. This is supported by the consistent results of earlier studies emphasizing that TPB-based interventions in physical activity are associated with positive changes in attitude (Chatzisarantis and Hagger, 2005; Parrott et al., 2008; Sanaeinasab et al., 2020).

### Limitations and Future Research

This study had several limitations. First, the SDT constructs were summarized into BPNS and RAI to ensure the simplicity of the research model. Consideration of the individual influence of the detailed BPNS (autonomy, competence, and relatedness) and RAI (autonomous/controlled motivation) indicators on social cognitive beliefs could have presented more diverse results (Chemolli and Gagné, 2014). Similarly, TPB, including belief variables, could be further segmented through an elicitation study for the composition of beliefs related to the target behavior (physical activity) in a specific context (COVID-19), as suggested by Ajzen (1991, 2002). Next, this study focused only on social and cognitive mechanisms, and failed to consider actual physical activities set as target behaviors. This was a choice made by the researchers based on the judgment that it was inappropriate to set intention-behavior variables to be measured simultaneously as a causal relationship according to the cross-sectional study design. However, in the demographics of the study participants, the proportion of the South Korean population that met the standard recommended by the WHO (MVPA 150 min./week), has clearly decreased since the pandemic began (50.2 → 29.2%). Therefore, it would be better to include longitudinal data such as the rate of change of target behavior to ensure the predictive validity of the study (Rhodes and Rebar, 2018). Finally, the sample of the current study focused on men in their 20 and 30s. Moreover, the type of person who would be willing to participate in an online platform such as Google Survey may not be typical of the population. Thus, it is difficult to sufficiently represent the South Korean population. For future research, it would be necessary to consider the dynamic interactions between demographic variables to understand the more specific effects of the included covariates.

## Conclusion

Globally, it is an unprecedented time. Surviving COVID-19 has become a challenge for humanity. Although a vaccine for COVID-19 has been developed and gradually spread from the beginning of this year, many countries are still responding to it through continuous distancing, isolation, and quarantine measures. The emergence of a highly infectious coronavirus in a mutated form, which has been reported recently, raises concerns about a prolonged pandemic (European Centre for Disease Prevention Control, 2020). At this point, limited opportunities to participate in physical activity have been considered a public health issue related to physical or mental damage to individuals, which can cause vulnerability to COVID-19. The current study identified that satisfaction with basic psychological needs predicts the level of self-determinant motivation for physical activity, which is partially related to the level of social cognitive beliefs and intentions. This study adds to the expanding body of knowledge revealing that using the integrated model of SDT and TPB can explain people's participation in physical activity during COVID-19. In particular, the mediating effect of attitude was the most significant. The findings highlight that the approach to emphasize a positive attitude toward physical activity would be a useful intervention in solving those problems.

## Data Availability Statement

The raw data supporting the conclusions of this article will be made available by the authors, without undue reservation.

## Ethics Statement

The studies involving human participants were reviewed and approved by the Institutional Review Board of the Seoul National University. The patients/participants provided their written informed consent to participate in this study.

## Author Contributions

DJ performed the analysis and interpretation of data and wrote the manuscript with input from all authors. IK provided critical feedback and helped shape the research, analysis, and manuscript. SK designed and directed the project. All authors have read and approved the final version of the manuscript and agree with the order of presentation of the authors.

## Conflict of Interest

The authors declare that the research was conducted in the absence of any commercial or financial relationships that could be construed as a potential conflict of interest.

## Publisher's Note

All claims expressed in this article are solely those of the authors and do not necessarily represent those of their affiliated organizations, or those of the publisher, the editors and the reviewers. Any product that may be evaluated in this article, or claim that may be made by its manufacturer, is not guaranteed or endorsed by the publisher.
