# Supplementary material for: Motivation and Intention Toward Physical Activity During the COVID-19 Pandemic: Perspectives From Integrated Model of Self-Determination and Planned Behavior Theories
Source: Front Psychol. 2021 Jul 29;12:714865. doi: 10.3389/fpsyg.2021.714865 (PMC8358808; doi:10.3389/fpsyg.2021.714865)
Supplement: Supplementary file 1 [file Data_Sheet_1.docx]

**Appendix. Final Measurement Model’s Factor Loadings**

| ***Construct*** | ***Items*** | | ***Loadings*** |
| --- | --- | --- | --- |
| **Autonomy** | 1. I feel free to exercise in my own way during the COVID-19 pandemic.  2. I feel free to make my own exercise program decisions during the COVID-19 pandemic.  3. I feel like I am in charge of my own exercise program decisions during the COVID-19 pandemic.  4. I feel like I have a say in choosing the exercises that I do during the COVID-19 pandemic.  5. I feel free to choose which exercises I participate during the COVID-19 pandemic.  6. I feel like I am the one who decides what exercises I do during the COVID-19 pandemic. | | .890  .907  .791  -  -  - |
| **Competence** | 1. I feel that I am able to complete exercises that are personally challenging during the COVID-19 pandemic.  2. I feel confident I can do even the most challenging exercises during the COVID-19 pandemic.  3. I feel confident in my ability to perform exercises that are personally challenging to me during the COVID-19 pandemic.  4. I feel capable of competing exercises that are challenging to me during the COVID-19 pandemic.  5. I feel like I am capable of doing even the most challenging exercises during the COVID-19 pandemic.  6. I feel good about the way I am able to complete challenging exercises during the COVID-19 pandemic. | | -  .919  .908  -  .920  - |
| **Relatedness** | 1. I feel attached to my exercise companions during the COVID-19 pandemic because they accept me for who I am.  2. I feel like I share a common bond with people who are important to me when we exercise during the COVID-19 pandemic.  3. I feel sense of camaraderie with my exercise companions because we exercise for the same reasons during the COVID-19 pandemic.  4. I feel close to my exercise companions who appreciate how difficult exercise can be during the COVID-19 pandemic.  5. I feel connected to the people who I interact with while we exercise during the COVID-19 pandemic.  6. I feel like I get along well with other people who I interact with while we exercise during the COVID-19 pandemic. | | -  .929  .951  -  .853  - |
| **Autonomous motivation** | In the COVID-19 pandemic, I (will) participate in physical activity,  because... | 1. Exercise is fun.  2. I enjoy my exercise sessions.  3. I find exercise a pleasurable activity.  4. I get pleasure and satisfaction from participating in exercise. | -  .874  .923  .908 |
| **Controlled motivation** |  | 1. Other people say I should.  2. My friends/family/partner say I should.  3. Others will not be pleased with me if I don’  4. I feel under pressure from my friends/family to exercise. | .825  .767  -  .762 |
| **Attitude** | 1. Exercising during the COVID-19 pandemic is valuable and meaningful to me.  2. Being physically active during the COVID-19 pandemic is beneficial to my health.  3. Exercise during the COVID-19 pandemic in useful and interesting to me.  4. Being physically active during the COVID-19 pandemic makes me feel good and enjoyable. | | .826  .831  .922  .866 |
| **Subjective norm** | 1. Other people continuously recommend that I get physically active during the COVID-19 pandemic.  2. Most others support me to exercise during the COVID-19 pandemic.  3. Someone important to me encourages me to exercise during the COVID-19 pandemic.  4. Other people around me engage in regular physical activity during the COVID-19 pandemic. | | .768  .875  .841  .765 |
| **Perceived behavioral control** | 1. I am confident to exercise regularly during the COVID-19 pandemic.  2. I have physical abilities to be physically active during the COVID-19 pandemic.  3. I do or get physically active whenever and wherever (I want to) during the COVID-19 pandemic.  4. I feel in complete control over whether I will exercise during the COVID-19 pandemic. | | .909  .766  .805  .728 |
| **Intention** | 1. I will participate in various physical activities within the next month.  2. I am planning to exercise regularly within the next month.  3. I will participate in sports or physical activities within the next month.  4. I will not exercise or engage in physical activities in leisure times. (R) | | .749  .939  .934  - |
